# Mitral Valve Lymphoma: An Unusual Cause of Embolic Strokes

**DOI:** 10.1016/j.atssr.2025.09.028

**Published:** 2025-10-29

**Authors:** Atharv Oak, Abdullah Arain, Uzma Rahman, Sumera I. Yilmaz, Jesse Suben, Ahmet Kilic

**Affiliations:** 1 The Johns Hopkins University School of Medicine, Baltimore, Maryland; 2 Department of General Surgery, Johns Hopkins Hospital, Baltimore, Maryland; 3 Division of Cardiac Surgery, Johns Hopkins Hospital, Baltimore, Maryland; 4 Bezmialem Vakif University of Medicine, Istanbul, Turkey; 5 Department of Pathology, Johns Hopkins Hospital, Baltimore, Maryland

## Abstract

An 80-year-old woman with a history of non-Hodgkin lymphoma and branch retinal artery occlusion presented with acute visual changes and received a diagnosis of multifocal embolic cerebral infarcts. Cardiac imaging revealed a mobile left atrial mass on the mitral valve. Surgical excision was undertaken because of the high risk of recurrent embolization and strokes. Histopathologic examination demonstrated chronic lymphocytic leukemia/small lymphocytic lymphoma within a fibrinous and calcified matrix. This case illustrates a rare intracardiac manifestation of lymphoma serving as a cardioembolic source and highlights the importance of considering hematologic malignant disease in the differential diagnosis of intracardiac masses.

Cardiac tumors are rare entities, with secondary cardiac tumors more common than primary cardiac tumors.^1^ These tumors are frequently discovered incidentally on imaging but can be symptomatic depending on their size and location. A comprehensive approach using multiple imaging modalities is often essential for accurate diagnosis and management of these lesions. Among secondary cardiac tumors, lymphomas are rare, comprising 1% of these tumors.^2^^,^^3^ They can be classified as either primary cardiac lymphoma (PCL) or secondary cardiac lymphoma (SCL). PCL arises predominantly in the heart or pericardium, with minimal or no extracardiac involvement at diagnosis. SCL occurs in the setting of systemic lymphoma, usually as part of advanced-stage disease. These tumors may manifest with nonspecific cardiac symptoms such as heart failure, arrhythmias, pericardial effusion, cardiac masses, or myocardial infiltration.^4^ Although thromboembolic phenomena can be associated with various cardiac tumors, cardiac lymphoma as a cause of stroke has been mentioned only in limited case reports.^5^ Here we present a case of a patient with cardioembolic strokes originating from lymphoma on the mitral valve and requiring surgical resection.

An 80-year-old woman with a previous history of stroke and intraabdominal lymphoma, who was receiving treatment with zanubrutinib, presented with blurry vision. She denied any weakness in an extremity, dysarthria, chest pain, shortness of breath, night sweats, or weight loss. She had no neurologic deficits on physical examination. A computed tomographic (CT) scan of the head had negative results for any acute infarct. However, a magnetic resonance imaging (MRI) scan of her brain revealed multiple subcentimeter infarcts involving the bilateral cerebral hemispheres and the right cerebellar hemisphere, findings consistent with a cardioembolic source. Results of carotid duplex studies were negative for any hemodynamically significant stenosis or occlusion. A transthoracic echocardiogram (TTE) showed a normal ejection fraction of 60% with no wall motion or valvular abnormalities. However, an echo-dense mass or thrombus in the left atrium was noted. The TTE was followed up with a transesophageal echocardiogram (TEE), which showed a 1.0 cm × 1.1 cm mobile, pedunculated, well-circumscribed cystic mass just below the level of the posterior mitral leaflet insertion. A CT scan of the chest, abdomen, and pelvis showed generalized lymphadenopathy, which was consistent with her known history of lymphoma. The patient was started on a heparin infusion, and a multidisciplinary discussion was held. Given that she was at high risk for recurrent embolic strokes in the setting of a cardiac mass, the decision was made to resect the mass surgically for diagnostic and therapeutic purposes.

An intraoperative TEE showed a dilated left atrium, with a 1.2 cm × 1 cm mass adherent to the posterior mitral annulus (Video, Figure 1A). The P2 segment had reduced excursion because of the mass. The mitral valve annulus appeared calcified (Figure 1B), with mild mitral regurgitation. We approached this mass through a median sternotomy. The patient was placed on cardiopulmonary bypass using ascending aortic and bicaval cannulation. Antegrade del Nido cardioplegia was used. The left atrium was entered through the Sondergaard groove, and the mitral valve was visualized. There were significant calcifications along the annulus, and the lesion was noted on the P2 segment, consistent with the TEE findings. This lesion was highly mobile, with caseous necrosis and a stalklike appearance. This lesion was excised in its entirety without violating or resecting the mitral valve leaflet and was sent for pathologic examination. Intraoperative cultures were sent to rule out subclinical endocarditis.

**Figure 1:**
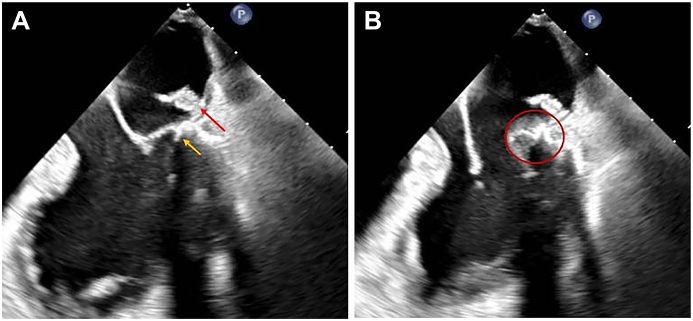
(A) Red and yellow arrows denote the mobile mass and the P2 segment, respectively. (B) The circle denotes the calcified mitral valve annulus.

The underlying mitral valve in this region was hyperemic and inflammatory. The atriotomy was closed with running 3-0 polypropylene (Prolene, Ethicon) suture with pledgets. The patient was weaned from cardiopulmonary bypass, and the rest of the case proceeded uneventfully. A TEE showed no change in mitral valve function. The patient had an uneventful postoperative course and was discharged home on postoperative day 7, with rivaroxaban. Pathologic findings were was consistent with chronic lymphocytic leukemia/small lymphocytic lymphoma in a background of abundant fibrinous debris and dystrophic calcifications (Figure 2). Results of tissue cultures for bacteria, fungi, and acid-fast bacilli were negative.

**Figure 2:**
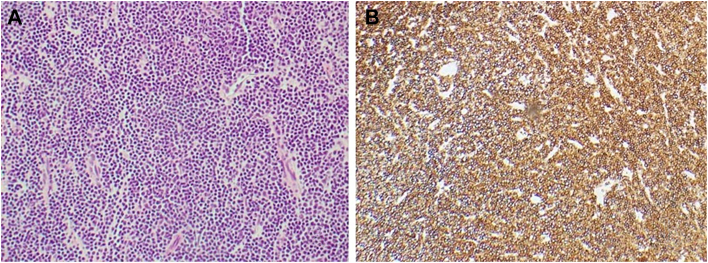
(A) Dense atypical lymphoid infiltrate composed of small, monotonous, mature lymphocytes. (B) Diffusely positive CD20 immunohistochemical stain (B-lymphocyte marker).

## Comment

Cardiac lymphomas are rare entities and most commonly occur in the setting of systemic disease. Non-Hodkin lymphoma is the most common type of cardiac lymphoma, for both PCL and SCL. Cardiac involvement in SCL is a sequela of hematogenous or lymphatic spread or of direct extension in the case of mediastinal tumors. When symptoms are present, they include heart failure, arrhythmias, and superior vena cava syndrome. Multimodal cardiac imaging is required for diagnosis and should be tailored to the patient’s presentation and history.

Although a cardiac mass can be identified on an echocardiogram with reliable sensitivity, TTE or TEE is usually not sufficient to differentiate a lymphoma from other cardiac masses, such as a thrombus, vegetation, or myxoma. Cardiac MRI is an excellent adjunct to characterize cardiac masses and evaluate the degree of involvement. Positron emission tomography combined with CT has also been reported to be a useful imaging modality in diagnosing cardiac lymphomas and monitoring response to treatment. Treatment is usually aimed at targeting the underlying disease, primarily with systemic chemotherapy. However, surgical resection can be considered for diagnosis or symptomatic relief.^6^ We determined that, in our patient, additional imaging with cardiac MRI or a positron emission tomographic scan would offer limited benefit and cause delay in treatment. Regardless of the origin or type of cardiac mass, it was most likely the source of her stroke, and awaiting additional imaging tests would only expose her to more thromboembolic events. Therefore, we decided that surgically excising this mass would be both diagnostic and therapeutic. The patient is now more than a year out from the surgical procedure, without any new episodes of stroke.

In conclusion, cardiac lymphomas are rare entities and can manifest with embolic strokes. Although cardiac lymphomas are usually treated with chemotherapy, surgical resection should be considered for diagnostic and therapeutic purposes in select patients.
